# Supplementary figures and images for: Heading towards a dead end: The role of DND1 in germ line differentiation of human iPSCs
Source: PLoS One. 2021 Oct 15;16(10):e0258427. doi: 10.1371/journal.pone.0258427 (PMC8519482; doi:10.1371/journal.pone.0258427)

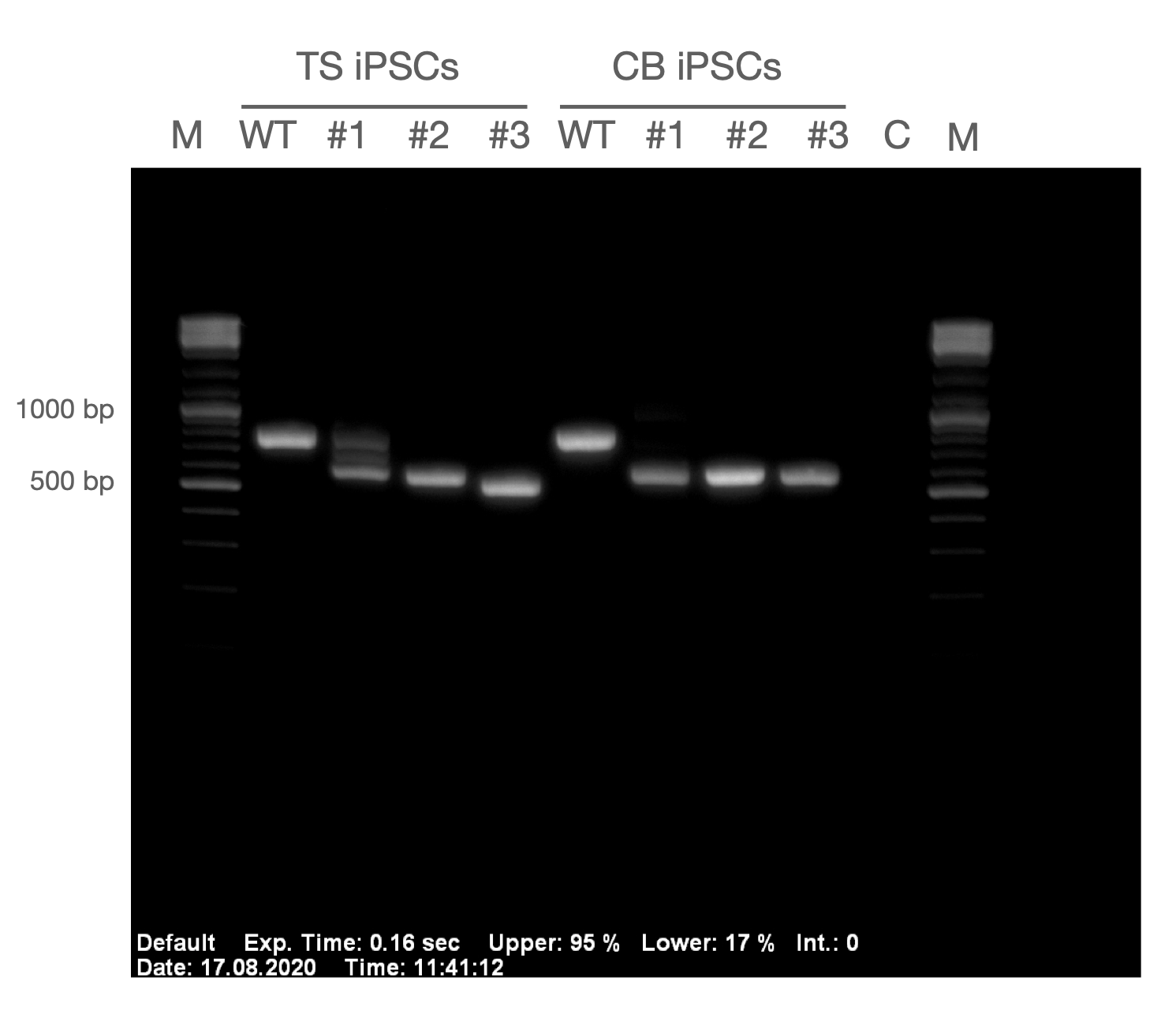

Supplement: S1 Raw images — (TIFF) [file pone.0258427.s001.tiff]
