# Supplementary material for: Heading towards a dead end: The role of DND1 in germ line differentiation of human iPSCs
Source: PLoS One. 2021 Oct 15;16(10):e0258427. doi: 10.1371/journal.pone.0258427 (PMC8519482; doi:10.1371/journal.pone.0258427)
Supplement: S1 File — (DOCX) [file pone.0258427.s002.docx]

**Heading towards a dead end: the role of *DND1* in germ line differentiation of human iPSCs**

Eva M. Mall^1,2^, Aaron Lecanda^1^, Hannes Drexler^1^, Erez Raz^3^*,* Hans R. Schöler^1^, Stefan Schlatt^2^

^1^ Max Planck Institute for Molecular Biomedicine, Röntgenstraße 20, 48149 Münster, Germany.

^2^ Centre of Reproductive Medicine and Andrology, Albert-Schweitzer-Campus 1, 48149 Münster, Germany.

^3^ Institute of Cell Biology, ZMBE, Von-Esmarch-Straße 56, 48149 Münster, Germany

Running title: DND1 in human iPSCs

Keywords: DND1, human pluripotent stem cells, primordial germ cells, differentiation


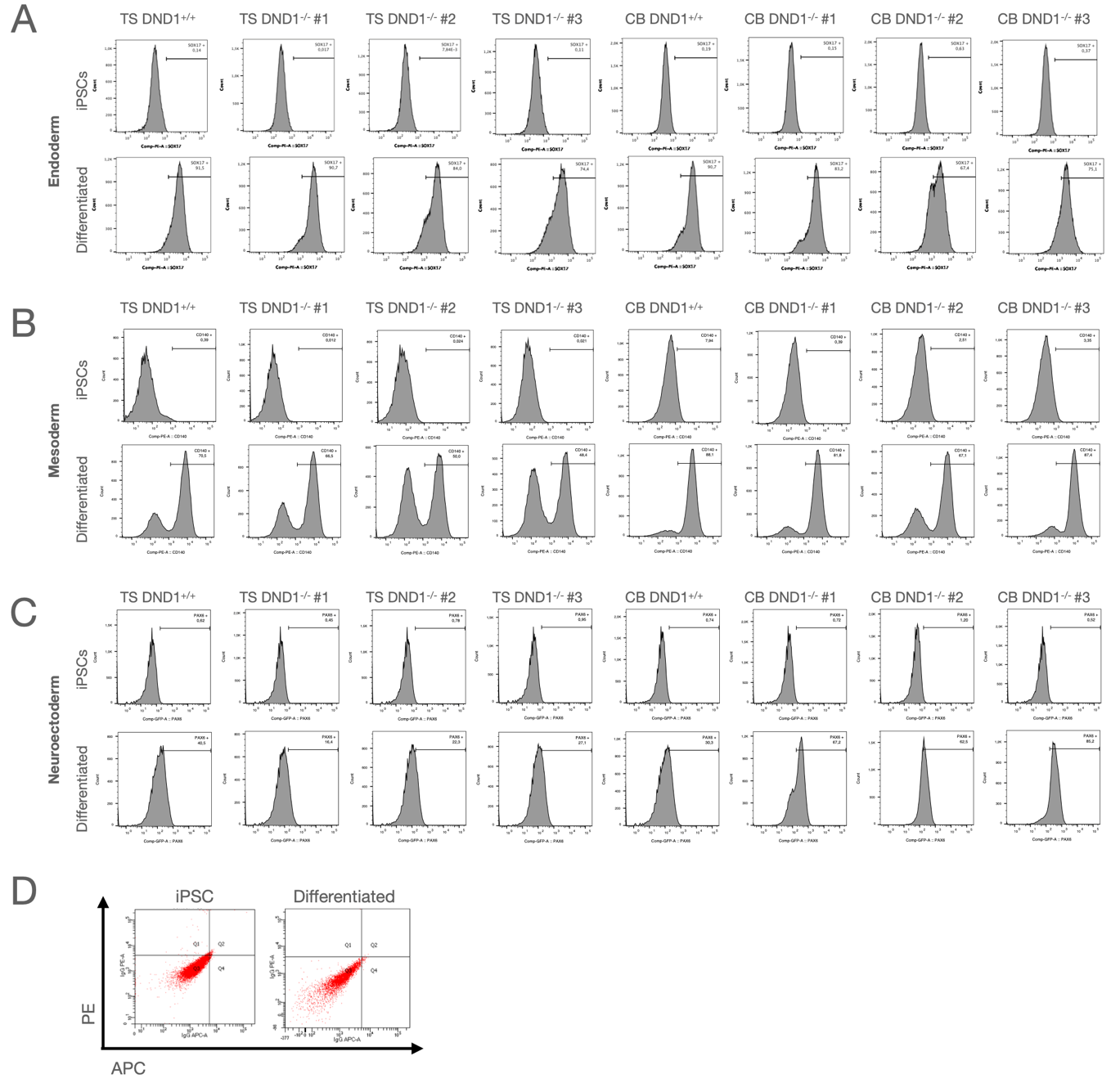
Supplemental Figure 1: Flow cytrometric analysis of trilineage differentiation. All lines were differentiated towards mesoderm (A, CD140), endoderm (B, SOX17), and neuroectoderm (C, PAX6). For each line and staining, undifferentiated iPSCs are shown as negative controls. Panel (D) depicts control staining with isotype match IgGs (PE and APC).


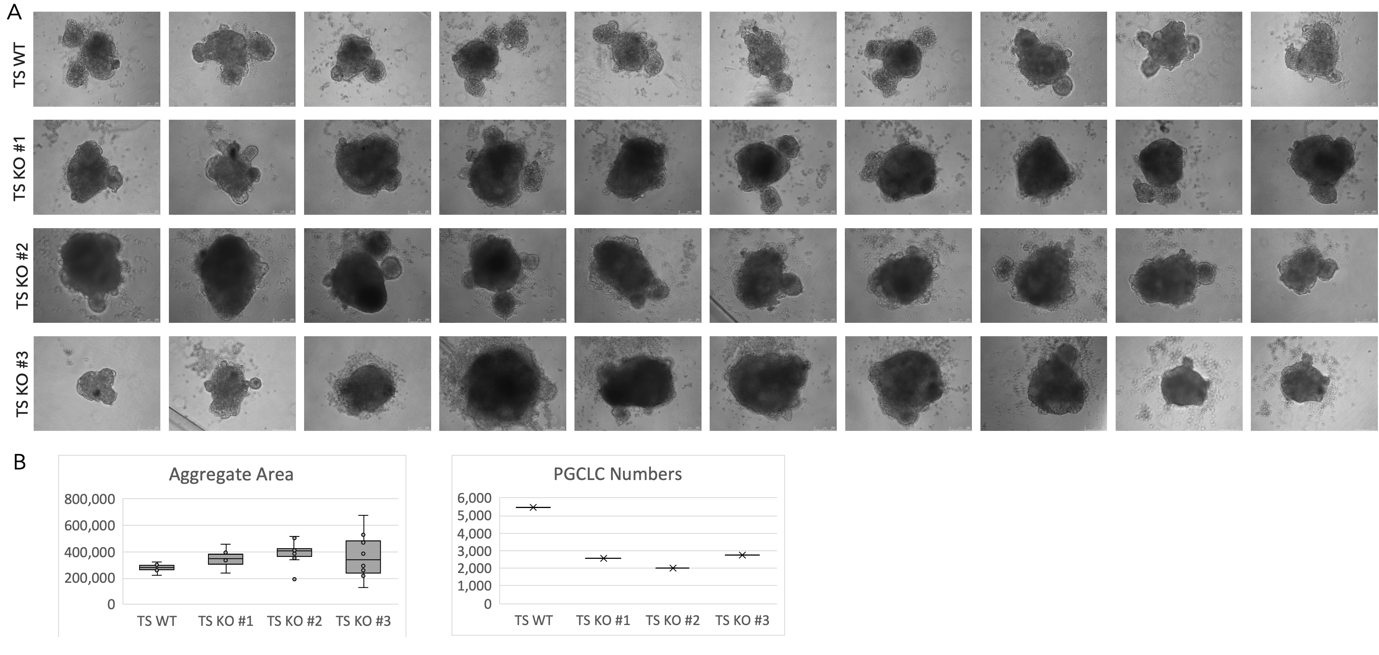
Supplemental Figure 2: EB sizes in TS iPSCs. (A) TS iPSCs were differentiated towards PGCLCs in 96-well low adhesion plates and aggregate sizes were measured at D5. (n = 1 experiment, 10 aggregates measured) (B) Box plots of different areas show increased sizes in DND1-/- aggregates, while the number of derived PGCLCs was reduced (n=1 experiment, one 96 well plate per line)

Supplemental Figure 3: RNAseq data comparison of generated data set from WT cells with published data from Irie et al. Left: PCA plot indicating different clusters. Right: GO term analysis of genes expressed higher or lower in Irie-PGCLCs compared to our PGCLCs.
