# Supplementary material for: Heading towards a dead end: The role of DND1 in germ line differentiation of human iPSCs
Source: PLoS One. 2021 Oct 15;16(10):e0258427. doi: 10.1371/journal.pone.0258427 (PMC8519482; doi:10.1371/journal.pone.0258427)
Supplement: S2 File — (DOCX) [file pone.0258427.s003.docx]

**TS KO #1**

TATACTAACCAGGGGTTTAATATAAATACAACCAGCATAGAAAGACCCAAAACTATACAGAAACCAAAACCAGAATGCCATGTGGTGGAGGCAAAGGGCAGAATTTCTGACCCCTTTGGCTCAGCTGCCCTTCCCCACAAATAAAAACCAACAAAGAGGACAAATCAGGACAATAAAGAAGATTCATGCTAAGCTGTGGCAGAGGGGGGAAGGTATGATCGGGTGGGGGTGGGACAAGGAACGGCCATGGAAGATCACTGGGTCAGGTTGGACCCTGGGCTGGGAGCGGGAGGGCAAGGCCCCTCACCACAACTTAACCCAAACCTAAGCTGCCCCCAGGTGCCATAGGTCCCTGTCCCAGCAGGGAGGCTGATGGGCCTGGGCCCATGCCCCTCCCCACCTTTGGGGGTCAGAAAGTGGCCACCCAGGCCTGCTGGGATGGGGCCTGACACAGGCTCTGCATGCCCATTCAGGGTGCCTGTGGAGAAAGAATGGAGTCACTGTTTAACCATGGTACCTGCCTCAGCCCCAGCAGACCACAGGAGTTGGCCCCAGACTCACTGAGTGCCTGCAGCAGCCGTACAGACACAGCATCCTTGGCCACCTCATGCCCATCCCGGCCATCTAGGGTCAGCACAACCCAGATGAGGCCGCTGAAGGGCACCGGATGCCCAGGAATCACCACCTGGTACCAGAAGCGGTGCCAGCCAGCAGGTCCTATGCCCAAACACTTGGTGAGGAACACAGGGCTGCCCAGCTTCATTCGTTGGCACAGCAACTGCAGGGTAGCCCGAGCCCCTTGGAACCCTAACTTGTCCCTTGCCAAAGCCAACTGGCTGCCCTCTGGCTGTGGGGACCGCAAGAAGGGACCCACAAGCTGCTGGCGAAGTCGCTGCTTCAGGTCTGGCTTGAGCCACTCCACAGCCACCTGCTCTCCACAGAGGTGTGACTGCCCTGTAGGAAAAATGCAAAGACAAGGGCAGGTCTAAACCCTGGGCCCAAGCCTCCAGGTGGTGAGCCCTTTGGAGCTACACAGTCCTGTTATTTGTAGCCTTCCCATTTCCTGGTAGTGTGACCATGGGTAAGAAAAGACAATGAAGCCTTCAGCCTCCATTATTTGCAAAGGGAGTATAACACACTGCTACCTTACAAGTTTCCTTGGAGAAGTACTGGTTAATTGCAGGCTTTGTCTGCCTCATAACCATCATAATGGCTAATCTTTACTGGGAAAACTTGCTGTAAGTCAGTCAGTGTGCTAAGTACCGTACACCCATTATGTTACTTAATTCTCATAACAGTCTGAGGAAACAGATTCTATAGTAGTAAAAAGCTCAGTTGGATTCCCTTGAGCAAATCAATTTCTCTAACAGTTTCCTCATAGCTTGAGGGTCCTGTGCCTATTTTGCGGTGTGGGGGTGGGGGAAGGTTGAGAATTAAGTCAGGAGGTAATGCTCGGGAAGTGTCACAAATTTAGGTAAGCGGTGGTGGTAGCACCATTGGAAGTTTTAAAAATCTGAGATCAAATAGTAAAAGGTTGCAAATTCTGGCATGTTTGACTCTAAGATCTTGTAACTTTAACAGCTATGCTCAGTATAAAGTGCCGGGCAGAACGCGTTTGGCCCCAACCAGCACCCCCGCCCCAGCCTACCTTCCACCAGGGCCTTTTTGGCCATGGCAGCGGCCCGGTGCGAGCTGAATTTGAGCAGAGCGATCTGCCCGGGCGCCGGTCCGGGGCTGGGCAGCAGCCGCGCCTCCTGCAAGCCGGGACCCAGCGGCTGCAGCGCGAGCAGCAGCGCGCTGCGGGTCAGATTCGGCGGCAGGCCGTCAACGCTCAGCTCACACTTCTCGGTGCTGCGGCACACGAGCAGCGGGCAGGACGGCCGCAGCGGATGGTTGTGCAGCGTGGCGATGGCGGCCTGCGCGCCGCGCCTCGAGCTGTAGCGGGCATAGGCGAAGCCGCGGTTCAGGCCGCTGAAGGTCATCATCAGGCGGAACTCGTAGAGGCGGCCCACGCGCTGGAACAGCGGGATAAGCTGGTGCTCGTACACGTCCTGAGGCAGCCGCCCGATGAACACCTCTGACCCAGCTGGCGGCGGGCTGCCCACCCAGCCTGTGGGAAGAGGGTATGCAAGGCCACCGTCAGGCGACGCTTTCGAATTCTGAGCCCACGGACCCAGCGCGCGGGGGTGGGAGGCGATGTGAACAAAGGTCTGTGAAATGGGTTTACACCCGTACCCTGGGTGGTTGGCATAATTAGGTGACTGCGCTAACAAGGGGGCTGGCACAGAGTAGGTACACCTTGAGATTGGCCCCCTCCCTCGACGGGGCGGGTGGACGTGGAGCCACAGTTCCTCCTTTGCTGGGGGGTATAAATCCGGGTAGTTCGCAGTTTCTGACAGTGAAGGCTCGGAGCAGCCCCTCCCCCAAAGGGGGCTGGTGTAGCCGGACAGGCGGAGGGGCTGGGACTACCGTACCTGGGGGTGGCCCGCCATACTTCCTCTGCCCGTTCACCTGCACGCTTGGACTGCATGGCTCTCCAGCTGGCCCCCTCGTACCCTCTTTATAACTTCCTCC

**TS KO #1**

ATACTAACCAGGGGTTTAATATAAATACAACCAGCATAGAAAGACCCAAAACTATACAGAAACCAAAACCAGAATGCCATGTGGTGGAGGCAAAGGGCAGAATTTCTGACCCCTTTGGCTCAGCTGCCCTTCCCCACAAATAAAAACCAACAAAGAGGACAAATCAGGACAATAAAGAAGATTCATGCTAAGCTGTGGCAGAGGGGGGAAGGTATGATCGGGTGGGGGTGGGACAAGGAACGGCCATGGAAGATCACTGGGTCAGGTTGGACCCTGGGCTGGGAGCGGGAGGGCAAGGCCCCTCACCACAACTTAACCCAAACCTAAGCTGCCCCCAGGTGCCATAGGTCCCTGTCCCAGCAGGGAGGCTGATGGGCCTGGGCCCATGCCCCTCCCCACCTTTGGGGGTCAGAAAGTGGCCACCCAGGCCTGCTGGGATGGGGCCTGACACAGGCTCTGCATGCCCATTCAGGGTGCCTGTGGAGAAAGAATGGAGTCACTGTTTAACCATGGTACCTGCCTCAGCCCCAGCAGACCACAGGAGGTTGGCCCCAGACTCACTGAGTGCCTGCAGCAGCCGTACAGACACAGCATCCTTGGCCACCTCATGCCCATCCCGGCCATCTAGGGTCAGCACAACCCAGATGAGGCCGCTGAAGGGCACCGGATGCCCAGGAATCACCACCTGGTACCAGAAGCGGTGCCAGCCAGCAGGTCCTATGCCCAAACACTTGGTGAGGAACACAGGGCTGCCCAGCTTCATTCGTTGGCACAGCAACTGCAGGGTAGCCCGAGCCCCTTGGAACCCTAACTTGTCCCTTGCCAAAGCCAACTGGCTGCCCTCTGGCTGTGGGGACCGCAAGAAGGGACCCACAAGCTGCTGGCGAAGTCGCTGCTTCAGGTCTGGCTTGAGCCACTCCACAGCCACCTGCTCTCCACAGAGGTGTGACTGCCCTGTAGGAAAAATGCAAAGACAAGGGCAGGTCTAAACCCTGGGCCCAAGCCTCCAGGTGGTGAGCCCTTTGGAGCTACACAGTCCTGTTATTTGTAGCCTTCCCATTTCCTGGTAGTGTGACCATGGGTAAGAAAAGACAATGAAGCCTTCAGCCTCCATTATTTGCAAAGGGAGTATAACACACTGCTACCTTACAAGTTTCCTTGGAGAAGTACTGGTTAATTGCAGGCTTTGTCTGCCTCATAACCATCATAATGGCTAATCTTTACTGGGAAAACTTGCTGTAAGTCAGTCAGTGTGCTAAGTACCGTACACCCATTATGTTACTTAATTCTCATAACAGTCTGAGGAAACAGATTCTATAGTAGTAAAAAGCTCAGTTGGATTCCCTTGAGCAAATCAATTTCTCTAACAGTTTCCTCATAGCTTGAGGGTCCTGTGCCTATTTTGCGGTGTGGGGGTGGGGGAAGGTTGAGAATTAAGTCAGGAGGTAATGCTCGGGAAGTGTCACAAATTTAGGTAAGCGGTGGTGGTAGCACCATTGGAAGTTTTAAAAATCTGAGATCAAATAGTAAAAGGTTGCAAATTCTGGCATGTTTGACTCTAAGATCTTGTAACTTTAACAGCTATGCTCAGTATAAAGTGCCGGGCAGAACGCGTTTGGCCCCAACCAGCACCCCCGCCCCAGCCTACCTTCCACCAGGGCCTTTTTGGCCATGGCAGCGGCCCGGTGCGAGCTGAATTTGAGCAGAGCGATCTGCCCGGGCGCCGGTCCGGGGCTGGGCAGCAGCCGCGCCTCCTGCAAGCCGGGACCCAGCGGCTGCAGCGCGAGCAGCAGCGCGCTGCGGGTCAGATTCGGCGGCAGGCCGTCAACGCTCAGCTCACACTTCTCGGTGCTGCGGCACACGAGCAGCGGGCAGGACGGCCGCAGCGGATGGTTGTGCAGCGTGGCGATGGCGGCCTGCGCGCCGCGCCTCGAGCTGTAGCGGGCATAGGCGAAGCCGCGGTTCAGGCCGCTGAAGGTCATCATCAGGCGGAACTCGTAGAGGCGGCCCACGCGCTGGAACAGCGGGATAAGCTGGTGCTCGTACACGTCCTGAGGCAGCCGCCCGATGAACACCTCTGACCCAGCTGGCGGCGGGCTGCCCACCCAGCCTGTGGGAAGAGGGTATGCAAGGCCACCGTCAGGCGACGCTTTCGAATTCTGAGCCCACGGACCCAGCGCGCGGGGGTGGGAGGCGATGTGAACAAAGGTCTGTGAAATGGGTTTACACCCGTACCCTGGGTGGTTGGCATAATTAGGTGACTGCGCTAACAAGGGGGCTGGCACAGAGTAGGTACACCTTGAGATTGGCCCCCTCCCTCGACGGGGCGGGTGGACGTGGAGCCACAGTTCCTCCTTTGCTGGGGGGTATAAATCCGGGTAGTTCGCAGTTTCTGACAGTGAAGGCTCGGAGCAGCCCCTCCCCCAAAGGGGGCTGGTGTAGCCGGACAGGCGGAGGGGCTGGGACTACCGTACCGCTTGGACTGCATGGCTCTCCAGCTGGCCCCCTCGTACCCTCTTTATAACTTCCTCC

**TS KO #3**

TATACTAACCAGGGGTTTAATATAAATACAACCAGCATAGAAAGACCCAAAACTATACAGAAACCAAAACCAGAATGCCATGTGGTGGAGGCAAAGGGCAGAATTTCTGACCCCTTTGGCTCAGCTGCCCTTCCCCACAAATAAAAACCAACAAAGAGGACAAATCAGGACAATAAAGAAGATTCATGCTAAGCTGTGGCAGAGGGGGGAAGGTATGATCGGGTGGGGGTGGGACAAGGAACGGCCATGGAAGATCACTGGGTCAGGTTGGACCCTGGGCTGGGAGCGGGAGGGCAAGGCCCCTCACCACAACTTAACCCAAACCTAAGCTGCCCCCAGGTGCCATAGGTCCCTGTCCCAGCAGGGAGGCTGATGGGCCTGGGCCCATGCCCCTCCCCACCTTTGGGGGTCAGAAAGTGGCCACCCAGGCCTGCTGGGATGGGGCCTGACACAGGCTCTGCATGCCCATTCAGGGTGCCTGTGGAGAAAGAATGGAGTCACTGTTTAACCATGGTACCTGCCTCAGCCCCAGCAGACCACAGGAGGTTGGCCCCAGACTCACTGAGTGCCTGCAGCAGCCGTACAGACACAGCATCCTTGGCCACCTCATGCCCATCCCGGCCATCTAGGGTCAGCACAACCCAGATGAGGCCGCTGAAGGGCACCGGATGCCCAGGAATCACCACCTGGTACCAGAAGCGGTGCCAGCCAGCAGGTCCTATGCCCAAACACTTGGTGAGGAACACAGGGCTGCCCAGCTTCATTCGTTGGCACAGCAACTGCAGGGTAGCCCGAGCCCCTTGGAACCCTAACTTGTCCCTTGCCAAAGCCAACTGGCTGCCCTCTGGCTGTGGGGACCGCAAGAAGGGACCCACAAGCTGCTGGCGAAGTCGCTGCTTCAGGTCTGGCTTGAGCCACTCCACAGCCACCTGCTCTCCACAGAGGTGTGACTGCCCTGTAGGAAAAATGCAAAGACAAGGGCAGGTCTAAACCCTGGGCCCAAGCCTCCAGGTGGTGAGCCCTTTGGAGCTACACAGTCCTGTTATTTGTAGCCTTCCCATTTCCTGGTAGTGTGACCATGGGTAAGAAAAGACAATGAAGCCTTCAGCCTCCATTATTTGCAAAGGGAGTATAACACACTGCTACCTTACAAGTTTCCTTGGAGAAGTACTGGTTAATTGCAGGCTTTGTCTGCCTCATAACCATCATAATGGCTAATCTTTACTGGGAAAACTTGCTGTAAGTCAGTCAGTGTGCTAAGTACCGTACACCCATTATGTTACTTAATTCTCATAACAGTCTGAGGAAACAGATTCTATAGTAGTAAAAAGCTCAGTTGGATTCCCTTGAGCAAATCAATTTCTCTAACAGTTTCCTCATAGCTTGAGGGTCCTGTGCCTATTTTGCGGTGTGGGGGTGGGGGAAGGTTGAGAATTAAGTCAGGAGGTAATGCTCGGGAAGTGTCACAAATTTAGGTAAGCGGTGGTGGTAGCACCATTGGAAGTTTTAAAAATCTGAGATCAAATAGTAAAAGGTTGCAAATTCTGGCATGTTTGACTCTAAGATCTTGTAACTTTAACAGCTATGCTCAGTATAAAGTGCCGGGCAGAACGCGTTTGGCCCCAACCAGCACCCCCGCCCCAGCCTACCTTCCACCAGGGCCTTTTTGGCCATGGCAGCGGCCCGGTGCGAGCTGAATTTGAGCAGAGCGATCTGCCCGGGCGCCGGTCCGGGGCTGGGCAGCAGCCGCGCCTCCTGCAAGCCGGGACCCAGCGGCTGCAGCGCGAGCAGCAGCGCGCTGCGGGTCAGATTCGGCGGCAGGCCGTCAACGCTCAGCTCACACTTCTCGGTGCTGCGGCACACGAGCAGCGGGCAGGACGGCCGCAGCGGATGGTTGTGCAGCGTGGCGATGGCGGCCTGCGCGCCGCGCCTCGAGCTGTAGCGGGCATAGGCGAAGCCGCGGTTCAGGCCGCTGAAGGTCATCATCAGGCGGAACTCGTAGAGGCGGCCCACGCGCTGGAACAGCGGGATAAGCTGGTGCTCGTACACGTCCTGAGGCAGCCGCCCGATGAACACCTCTGACCCAGCTGGCGGCGGGCTGCCCACCCAGCCTGTGGGAAGAGGGTATGCAAGGCCACCGTCAGGCGACGCTTTCGAATTCTGAGCCCACGGACCCAGCGCGCGGGGGTGGGAGGCGATGTGAACAAAGGTCTGTGAAATGGGTTTACACCCGTACCCTGGGTGGTTGGCATAATTAGGTGACTGCGCTAACAAGGGGGCTGGCACAGAGTAGGTACACCTTGAGATTGGCCCCCTCCCTCGACGGGGCGGGTGGACGTGGAGCCACAGTTCCTCCTTTGCTGGGGGGTATAAATCCGGGTAGTTCGCAGTTTCTGACAGTGAAGGCTCGGAGCAGCCCCTCCCCCAAAGGGGGCTGGTGTAGCCGGACAGGCGGAGGGGCTGGGACTACCGTACCGCTTGGACTGCATGGCTCTCCAGCTGGCCCCCTCGTACCCTCTTTATAACTTCCTCC

**CB KO #1**

TATACTAACCAGGGGTTTAATATAAATACAACCAGCATAGAAAGACCCAAAACTATACAGAAACCAAAACCAGAATGCCATGTGGTGGAGGCAAAGGGCAGAATTTCTGACCCCTTTGGCTCAGCTGCCCTTCCCCACAAATAAAAACCAACAAAGAGGACAAATCAGGACAATAAAGAAGATTCATGCTAAGCTGTGGCAGAGGGGGGAAGGTATGATCGGGTGGGGGTGGGACAAGGAACGGCCATGGAAGATCACTGGGTCAGGTTGGACCCTGGGCTGGGAGCGGGAGGGCAAGGCCCCTCACCACAACTTAACCCAAACCTAAGCTGCCCCCAGGTGCCATAGGTCCCTGTCCCAGCAGGGAGGCTGATGGGCCTGGGCCCATGCCCCTCCCCACCTTTGGGGGTCAGAAAGTGGCCACCCAGGCCTGCTGGGATGGGGCCTGACACAGGCTCTGCATGCCCATTCAGGGTGCCTGTGGAGAAAGAATGGAGTCACTGTTTAACCATGGTACCTGCCTCAGCCCCAGCAGACCACAGGAGGTTGGCCCCAGACTCACTGAGTGCCTGCAGCAGCCGTACAGACACAGCATCCTTGGCCACCTCATGCCCATCCCGGCCATCTAGGGTCAGCACAACCCAGATGAGGCCGCTGAAGGGCACCGGATGCCCAGGAATCACCACCTGGTACCAGAAGCGGTGCCAGCCAGCAGGTCCTATGCCCAAACACTTGGTGAGGAACACAGGGCTGCCCAGCTTCATTCGTTGGCACAGCAACTGCAGGGTAGCCCGAGCCCCTTGGAACCCTAACTTGTCCCTTGCCAAAGCCAACTGGCTGCCCTCTGGCTGTGGGGACCGCAAGAAGGGACCCACAAGCTGCTGGCGAAGTCGCTGCTTCAGGTCTGGCTTGAGCCACTCCACAGCCACCTGCTCTCCACAGAGGTGTGACTGCCCTGTAGGAAAAATGCAAAGACAAGGGCAGGTCTAAACCCTGGGCCCAAGCCTCCAGGTGGTGAGCCCTTTGGAGCTACACAGTCCTGTTATTTGTAGCCTTCCCATTTCCTGGTAGTGTGACCATGGGTAAGAAAAGACAATGAAGCCTTCAGCCTCCATTATTTGCAAAGGGAGTATAACACACTGCTACCTTACAAGTTTCCTTGGAGAAGTACTGGTTAATTGCAGGCTTTGTCTGCCTCATAACCATCATAATGGCTAATCTTTACTGGGAAAACTTGCTGTAAGTCAGTCAGTGTGCTAAGTACCGTACACCCATTATGTTACTTAATTCTCATAACAGTCTGAGGAAACAGATTCTATAGTAGTAAAAAGCTCAGTTGGATTCCCTTGAGCAAATCAATTTCTCTAACAGTTTCCTCATAGCTTGAGGGTCCTGTGCCTATTTTGCGGTGTGGGGGTGGGGGAAGGTTGAGAATTAAGTCAGGAGGTAATGCTCGGGAAGTGTCACAAATTTAGGTAAGCGGTGGTGGTAGCACCATTGGAAGTTTTAAAAATCTGAGATCAAATAGTAAAAGGTTGCAAATTCTGGCATGTTTGACTCTAAGATCTTGTAACTTTAACAGCTATGCTCAGTATAAAGTGCCGGGCAGAACGCGTTTGGCCCCAACCAGCACCCCCGCCCCAGCCTACCTTCCACCAGGGCCTTTTTGGCCATGGCAGCGGCCCGGTGCGAGCTGAATTTGAGCAGAGCGATCTGCCCGGGCGCCGGTCCGGGGCTGGGCAGCAGCCGCGCCTCCTGCAAGCCGGGACCCAGCGGCTGCAGCGCGAGCAGCAGCGCGCTGCGGGTCAGATTCGGCGGCAGGCCGTCAACGCTCAGCTCACACTTCTCGGTGCTGCGGCACACGAGCAGCGGGCAGGACGGCCGCAGCGGATGGTTGTGCAGCGTGGCGATGGCGGCCTGCGCGCCGCGCCTCGAGCTGTAGCGGGCATAGGCGAAGCCGCGGTTCAGGCCGCTGAAGGTCATCATCAGGCGGAACTCGTAGAGGCGGCCCACGCGCTGGAACAGCGGGATAAGCTGGTGCTCGTACACGTCCTGAGGCAGCCGCCCGATGAACACCTCTGACCCAGCTGGCGGCGGGCTGCCCACCCAGCCTGTGGGAAGAGGGTATGCAAGGCCACCGTCAGGCGACGCTTTCGAATTCTGAGCCCACGGACCCAGCGCGCGGGGGTGGGAGGCGATGTGAACAAAGGTCTGTGAAATGGGTTTACACCCGTACCCTGGGTGGTTGGCATAATTAGGTGACTGCGCTAACAAGGGGGCTGGCACAGAGTAGGTACACCTTGAGATTGGCCCCCTCCCTCGACGGGGCGGGTGGACGTGGAGCCACAGTTCCTCCTTTGCTGGGGGGTATAAATCCGGGTAGTTCGCAGTTTCTGACAGTGAAGGCTCGGAGCAGCCCCTCCCCCAAAGGGGGCTGGTGTAGCCGGACAGGCGGAGGGGCTGGGACTACCGTACCTGGGGGTGGCCCGCCATACTTCCTCTGCCCGTTCACCTGCACCGCTTGGACTGCATGGCTCTCCAGCTGGCCCCCTCGTACCCTCTTTATAACTTCCTCC

**CB KO #2**

TATACTAACCAGGGGTTTAATATAAATACAACCAGCATAGAAAGACCCAAAACTATACAGAAACCAAAACCAGAATGCCATGTGGTGGAGGCAAAGGGCAGAATTTCTGACCCCTTTGGCTCAGCTGCCCTTCCCCACAAATAAAAACCAACAAAGAGGACAAATCAGGACAATAAAGAAGATTCATGCTAAGCTGTGGCAGAGGGGGGAAGGTATGATCGGGTGGGGGTGGGACAAGGAACGGCCATGGAAGATCACTGGGTCAGGTTGGACCCTGGGCTGGGAGCGGGAGGGCAAGGCCCCTCACCACAACTTAACCCAAACCTAAGCTGCCCCCAGGTGCCATAGGTCCCTGTCCCAGCAGGGAGGCTGATGGGCCTGGGCCCATGCCCCTCCCCACCTTTGGGGGTCAGAAAGTGGCCACCCAGGCCTGCTGGGATGGGGCCTGACACAGGCTCTGCATGCCCATTCAGGGTGCCTGTGGAGAAAGAATGGAGTCACTGTTTAACCATGGTACCTGCCTCAGCCCCAGCAGACCACAGGAGGTTGGCCCCAGACTCACTGAGTGCCTGCAGCAGCCGTACAGACACAGCATCCTTGGCCACCTCATGCCCATCCCGGCCATCTAGGGTCAGCACAACCCAGATGAGGCCGCTGAAGGGCACCGGATGCCCAGGAATCACCACCTGGTACCAGAAGCGGTGCCAGCCAGCAGGTCCTATGCCCAAACACTTGGTGAGGAACACAGGGCTGCCCAGCTTCATTCGTTGGCACAGCAACTGCAGGGTAGCCCGAGCCCCTTGGAACCCTAACTTGTCCCTTGCCAAAGCCAACTGGCTGCCCTCTGGCTGTGGGGACCGCAAGAAGGGACCCACAAGCTGCTGGCGAAGTCGCTGCTTCAGGTCTGGCTTGAGCCACTCCACAGCCACCTGCTCTCCACAGAGGTGTGACTGCCCTGTAGGAAAAATGCAAAGACAAGGGCAGGTCTAAACCCTGGGCCCAAGCCTCCAGGTGGTGAGCCCTTTGGAGCTACACAGTCCTGTTATTTGTAGCCTTCCCATTTCCTGGTAGTGTGACCATGGGTAAGAAAAGACAATGAAGCCTTCAGCCTCCATTATTTGCAAAGGGAGTATAACACACTGCTACCTTACAAGTTTCCTTGGAGAAGTACTGGTTAATTGCAGGCTTTGTCTGCCTCATAACCATCATAATGGCTAATCTTTACTGGGAAAACTTGCTGTAAGTCAGTCAGTGTGCTAAGTACCGTACACCCATTATGTTACTTAATTCTCATAACAGTCTGAGGAAACAGATTCTATAGTAGTAAAAAGCTCAGTTGGATTCCCTTGAGCAAATCAATTTCTCTAACAGTTTCCTCATAGCTTGAGGGTCCTGTGCCTATTTTGCGGTGTGGGGGTGGGGGAAGGTTGAGAATTAAGTCAGGAGGTAATGCTCGGGAAGTGTCACAAATTTAGGTAAGCGGTGGTGGTAGCACCATTGGAAGTTTTAAAAATCTGAGATCAAATAGTAAAAGGTTGCAAATTCTGGCATGTTTGACTCTAAGATCTTGTAACTTTAACAGCTATGCTCAGTATAAAGTGCCGGGCAGAACGCGTTTGGCCCCAACCAGCACCCCCGCCCCAGCCTACCTTCCACCAGGGCCTTTTTGGCCATGGCAGCGGCCCGGTGCGAGCTGAATTTGAGCAGAGCGATCTGCCCGGGCGCCGGTCCGGGGCTGGGCAGCAGCCGCGCCTCCTGCAAGCCGGGACCCAGCGGCTGCAGCGCGAGCAGCAGCGCGCTGCGGGTCAGATTCGGCGGCAGGCCGTCAACGCTCAGCTCACACTTCTCGGTGCTGCGGCACACGAGCAGCGGGCAGGACGGCCGCAGCGGATGGTTGTGCAGCGTGGCGATGGCGGCCTGCGCGCCGCGCCTCGAGCTGTAGCGGGCATAGGCGAAGCCGCGGTTCAGGCCGCTGAAGGTCATCATCAGGCGGAACTCGTAGAGGCGGCCCACGCGCTGGAACAGCGGGATAAGCTGGTGCTCGTACACGTCCTGAGGCAGCCGCCCGATGAACACCTCTGACCCAGCTGGCGGCGGGCTGCCCACCCAGCCTGTGGGAAGAGGGTATGCAAGGCCACCGTCAGGCGACGCTTTCGAATTCTGAGCCCACGGACCCAGCGCGCGGGGGTGGGAGGCGATGTGAACAAAGGTCTGTGAAATGGGTTTACACCCGTACCCTGGGTGGTTGGCATAATTAGGTGACTGCGCTAACAAGGGGGCTGGCACAGAGTAGGTACACCTTGAGATTGGCCCCCTCCCTCGACGGGGCGGGTGGACGTGGAGCCACAGTTCCTCCTTTGCTGGGGGGTATAAATCCGGGTAGTTCGCAGTTTCTGACAGTGAAGGCTCGGAGCAGCCCCTCCCCCAAAGGGGGCTGGTGTAGCCGGACAGGCGGAGGGGCTGGGACTACCGTACCTGGGGGTGGCCCGCCATACTTCCTCTGCCCGTTCACCTGCACCGCTTGGACTGCATGGCTCTCCAGCTGGCCCCCTCGTACCCTCTTTATAACTTCCTCC

**CB KO #3**

TATACTAACCAGGGGTTTAATATAAATACAACCAGCATAGAAAGACCCAAAACTATACAGAAACCAAAACCAGAATGCCATGTGGTGGAGGCAAAGGGCAGAATTTCTGACCCCTTTGGCTCAGCTGCCCTTCCCCACAAATAAAAACCAACAAAGAGGACAAATCAGGACAATAAAGAAGATTCATGCTAAGCTGTGGCAGAGGGGGGAAGGTATGATCGGGTGGGGGTGGGACAAGGAACGGCCATGGAAGATCACTGGGTCAGGTTGGACCCTGGGCTGGGAGCGGGAGGGCAAGGCCCCTCACCACAACTTAACCCAAACCTAAGCTGCCCCCAGGTGCCATAGGTCCCTGTCCCAGCAGGGAGGCTGATGGGCCTGGGCCCATGCCCCTCCCCACCTTTGGGGGTCAGAAAGTGGCCACCCAGGCCTGCTGGGATGGGGCCTGACACAGGCTCTGCATGCCCATTCAGGGTGCCTGTGGAGAAAGAATGGAGTCACTGTTTAACCATGGTACCTGCCTCAGCCCCAGCAGACCACAGGAGGTTGGCCCCAGACTCACTGAGTGCCTGCAGCAGCCGTACAGACACAGCATCCTTGGCCACCTCATGCCCATCCCGGCCATCTAGGGTCAGCACAACCCAGATGAGGCCGCTGAAGGGCACCGGATGCCCAGGAATCACCACCTGGTACCAGAAGCGGTGCCAGCCAGCAGGTCCTATGCCCAAACACTTGGTGAGGAACACAGGGCTGCCCAGCTTCATTCGTTGGCACAGCAACTGCAGGGTAGCCCGAGCCCCTTGGAACCCTAACTTGTCCCTTGCCAAAGCCAACTGGCTGCCCTCTGGCTGTGGGGACCGCAAGAAGGGACCCACAAGCTGCTGGCGAAGTCGCTGCTTCAGGTCTGGCTTGAGCCACTCCACAGCCACCTGCTCTCCACAGAGGTGTGACTGCCCTGTAGGAAAAATGCAAAGACAAGGGCAGGTCTAAACCCTGGGCCCAAGCCTCCAGGTGGTGAGCCCTTTGGAGCTACACAGTCCTGTTATTTGTAGCCTTCCCATTTCCTGGTAGTGTGACCATGGGTAAGAAAAGACAATGAAGCCTTCAGCCTCCATTATTTGCAAAGGGAGTATAACACACTGCTACCTTACAAGTTTCCTTGGAGAAGTACTGGTTAATTGCAGGCTTTGTCTGCCTCATAACCATCATAATGGCTAATCTTTACTGGGAAAACTTGCTGTAAGTCAGTCAGTGTGCTAAGTACCGTACACCCATTATGTTACTTAATTCTCATAACAGTCTGAGGAAACAGATTCTATAGTAGTAAAAAGCTCAGTTGGATTCCCTTGAGCAAATCAATTTCTCTAACAGTTTCCTCATAGCTTGAGGGTCCTGTGCCTATTTTGCGGTGTGGGGGTGGGGGAAGGTTGAGAATTAAGTCAGGAGGTAATGCTCGGGAAGTGTCACAAATTTAGGTAAGCGGTGGTGGTAGCACCATTGGAAGTTTTAAAAATCTGAGATCAAATAGTAAAAGGTTGCAAATTCTGGCATGTTTGACTCTAAGATCTTGTAACTTTAACAGCTATGCTCAGTATAAAGTGCCGGGCAGAACGCGTTTGGCCCCAACCAGCACCCCCGCCCCAGCCTACCTTCCACCAGGGCCTTTTTGGCCATGGCAGCGGCCCGGTGCGAGCTGAATTTGAGCAGAGCGATCTGCCCGGGCGCCGGTCCGGGGCTGGGCAGCAGCCGCGCCTCCTGCAAGCCGGGACCCAGCGGCTGCAGCGCGAGCAGCAGCGCGCTGCGGGTCAGATTCGGCGGCAGGCCGTCAACGCTCAGCTCACACTTCTCGGTGCTGCGGCACACGAGCAGCGGGCAGGACGGCCGCAGCGGATGGTTGTGCAGCGTGGCGATGGCGGCCTGCGCGCCGCGCCTCGAGCTGTAGCGGGCATAGGCGAAGCCGCGGTTCAGGCCGCTGAAGGTCATCATCAGGCGGAACTCGTAGAGGCGGCCCACGCGCTGGAACAGCGGGATAAGCTGGTGCTCGTACACGTCCTGAGGCAGCCGCCCGATGAACACCTCTGACCCAGCTGGCGGCGGGCTGCCCACCCAGCCTGTGGGAAGAGGGTATGCAAGGCCACCGTCAGGCGACGCTTTCGAATTCTGAGCCCACGGACCCAGCGCGCGGGGGTGGGAGGCGATGTGAACAAAGGTCTGTGAAATGGGTTTACACCCGTACCCTGGGTGGTTGGCATAATTAGGTGACTGCGCTAACAAGGGGGCTGGCACAGAGTAGGTACACCTTGAGATTGGCCCCCTCCCTCGACGGGGCGGGTGGACGTGGAGCCACAGTTCCTCCTTTGCTGGGGGGTATAAATCCGGGTAGTTCGCAGTTTCTGACAGTGAAGGCTCGGAGCAGCCCCTCCCCCAAAGGGGGCTGGTGTAGCCGGACAGGCGGAGGGGCTGGGACTACCGTACCTGGGGGTGGCCCGCCATACTTCCTCTGCCCGTTCACCTGCACCAGGCGGATGCCTGTCTCCCTGACCCACGCCTCCAGCGCCGCCTTGTTCTCTGGATTCACCCTCTCACACCACAGCTGAGAGGGAAAGGAAGGTTGGAATGGCGGATCGCCAAGCGCGCCCCCACCTCTCCTGTGGTACTGGGGTCCCTAAAGCCGACCCCCGCTCCGGCGGGGCTCGCCGGCCCCCAAGTCGCCAGCCGCTTACCTCACAATCCCGCTTGGACTGCATGGCTCTCCAGCTGGCCCCCTCGTACCCTCTTTATAACTTCCTCC
